# Towards Rapid Fabrication of Superhydrophobic Surfaces by Multi-Beam Nanostructuring with 40,401 Beams

**DOI:** 10.3390/nano11081987

**Published:** 2021-08-02

**Authors:** Petr Hauschwitz, Radka Bičštová, Alexander Brodsky, Natan Kaplan, Martin Cimrman, Jaroslav Huynh, Jan Brajer, Danijela Rostohar, Jaromír Kopeček, Martin Smrž, Tomáš Mocek

**Affiliations:** 1 HiLASE Centre, Institute of Physics, Czech Academy of Sciences, Za Radnici 828, 252 41 Dolni Brezany, Czech Republic; bicistova@fzu.cz (R.B.); cimrman@fzu.cz (M.C.); huynh@fzu.cz (J.H.); jan.brajer@hilase.cz (J.B.); danijela.rostohar@hilase.cz (D.R.); smrz@fzu.cz (M.S.); mocek@fzu.cz (T.M.); 2 R & D Department, Holo/Or Ltd., Einstein 13b, Ness Tziona 7403617, Israel; brodsky@holoor.co.il (A.B.); natan@holoor.co.il (N.K.); 3 Faculty of Nuclear Sciences and Physical Engineering, Czech Technical University in Prague, Brehova 7, 115 19 Prague, Czech Republic; 4 Institute of Physics of the Czech Academy of Sciences, Na Slovance 2, 182 00 Prague, Czech Republic; kopecek@fzu.cz

**Keywords:** multi-beam processing, surface functionalization, superhydrophobicity, micromachining, diffractive optics, DOE

## Abstract

Superhydrophobic surfaces attract a lot of attention due to many potential applications including anti-icing, anti-corrosion, self-cleaning or drag-reduction surfaces. Despite a list of attractive applications of superhydrophobic surfaces and demonstrated capability of lasers to produce them, the speed of laser micro and nanostructuring is still low with respect to many industry standards. Up-to-now, most promising multi-beam solutions can improve processing speed a hundred to a thousand times. However, productive and efficient utilization of a new generation of kW-class ultrashort pulsed lasers for precise nanostructuring requires a much higher number of beams. In this work, we introduce a unique combination of high-energy pulsed ultrashort laser system delivering up to 20 mJ at 1030 nm in 1.7 ps and novel Diffractive Laser-Induced Texturing element (DLITe) capable of producing 201 × 201 sub-beams of 5 µm in diameter on a square area of 1 mm^2^. Simultaneous nanostructuring with 40,401 sub-beams resulted in a matrix of microcraters covered by nanogratings and ripples with periodicity below 470 nm and 720 nm, respectively. The processed area demonstrated hydrophobic to superhydrophobic properties with a maximum contact angle of 153°.

## 1. Introduction

The inspiration and design of functional surfaces may often come from nature by replicating examples including lotus or rice leaf, mosquito eye, shark skin or cicada wings [1,2]. Among many natural examples, superhydrophobic biological systems attract a lot of attention for applications including anti-icing, anti-corrosion, self-cleaning or drag-reduction surfaces [3], lab-on-chip devices [4], control of cellular attachment and bacterial adsorption [5].

The wettability of a given surface is characterized by the static contact angle (CA) which is the visually measurable angle that a liquid makes with a solid. The surface is hydrophilic for a CA below 90°, hydrophobic above this value. The surface can be called superhydrophobic when CA exceeds 150°. In general, the wetting conditions are described by the two factors: the chemical composition and the surface roughness, which both affect the surface free energy (SFE)—the work that would be necessary to increase the surface area of a solid phase. If the surface is ideally flat, the wettability is determined by the chemical nature of the different phases. In this case, the maximum CA is limited to ~120° [6,7]. To improve wettability further, roughness has to be introduced on the surface. The Wenzel and Cassie–Baxter theories explain wettability through surface roughness [8,9]. According to the Wenzel model describing a fully wetted surface, the originally hydrophilic surface will become more hydrophilic and originally hydrophobic more hydrophobic with the increase in surface roughness [10]. On the other hand, the Cassie–Baxter equation describes a rough surface, which is able to trap air between surface features when a droplet is deposited on the surface. In this case, water repellence can be enhanced for both, originally hydrophilic and hydrophobic surfaces.

A large variety of fabrication methods have been developed for fabrication of functional surfaces including chemical vapour deposition [11], lithography [12], chemical etching [13], plasma treatments [14] or laser surface texturing [15]. However, most of these techniques are too slow to be implemented in an industrial environment or they require chemicals and thus are not environmentally friendly. Among these, laser surface texturing offers a flexible, fast and environmentally friendly method for high-quality fabrication of desired micro and nano geometries with high precision and on a large variety of materials.

The high-precision laser fabrication of functional structures with dimensions below a few micrometres may often require maximum pulse energy and power to be close to the ablation threshold. In addition, close-to-threshold irradiation with ultrashort pulses may lead to the development of regular nanoscale structures known as Laser-induced Periodic Surface Structures (LIPSS), ripples or nanogratings, which may further enhance surface functionality [16,17,18]. As a result, only a small portion of available laser power is used during processing, making the potential industrial application expensive and less ecological.

One of the most promising solutions for industrial utilization of lasers for micromachining is multi-beam processing. In this solution, the incident beam is split into a matrix of sub-beams for simultaneous fabrication inside the optimal processing window [19]. This can be achieved by several techniques including Direct Laser Interference Patterning (DLIP) [20,21] and beam splitting by Diffractive Optical Element (DOE) [22] or Spatial Light Modulator (SLM) [23]. Employing up-to-date laser and optical technology these multi-beam approaches can reach high efficiency with high-energy pulsed laser systems by splitting the beam into hundreds of sub-beams or interference maxima positions. For example, Lang et al. [24] reached the world record in DLIP structuring of polymers with the throughput of 0.9 m^2^/min utilizing an elliptical interference area of 15,000 µm × 50 µm featuring ~676 interference maxima positions. In addition, more than 1000 individual microcraters were fabricated simultaneously by four beam interference patterning used by authors in previous work [25]. However, the common drawback for DLIP is interference maxima positions follow Gaussian distribution of the input laser beam. Therefore, slowing down patterning over a larger area due to required overlap of interference areas to achieve homogeneous pattern distribution [26].

More straightforward solution is the use of diffractive elements which may be applied directly in the optical setup. Moreover, advanced DOE design enables to split the beam into the square-shaped matrix of sub-beams with equal intensity. Thus, eliminating overlap for stitching over larger areas. Kuang et al. [27] demonstrated fast parallel microstructuring with 1 mJ femtosecond laser system utilizing 40 sub-beams in a rectangular matrix. Efficient micromachining with high power-ultrashort laser pulses and 144 sub-beams was also reported by Gillner et al. [28]. More than 784 sub-beams were applied for micromachining of invar in the previous work of authors [19]. However, to efficiently utilize the new generation of ultrashort laser systems with power above 0.5 kW and millijoule pulse energy [29] significantly higher number of sub-beams would be required for high-quality micro and nanostructuring together with a new type of DOEs.

This work introduces a novel method for rapid and cost-efficient production of superhydrophobic surfaces by utilizing a compact protype of processing optical system responsible for beam splitting and focusing. The optical system includes a new type of DOE capable of splitting the incident high-quality laser beam into the rectangular matrix of 201 × 201 sub-beams. By applying this optical system, a square-shaped area of 1 mm^2^ can be efficiently nanostructured during a few laser pulses by 40,401 sub-beams, which is, to the best of our knowledge, the highest number of sub-beams utilized for simultaneous mask-less laser nanostructuring.

## 2. Materials and Methods

**Figure 1:**
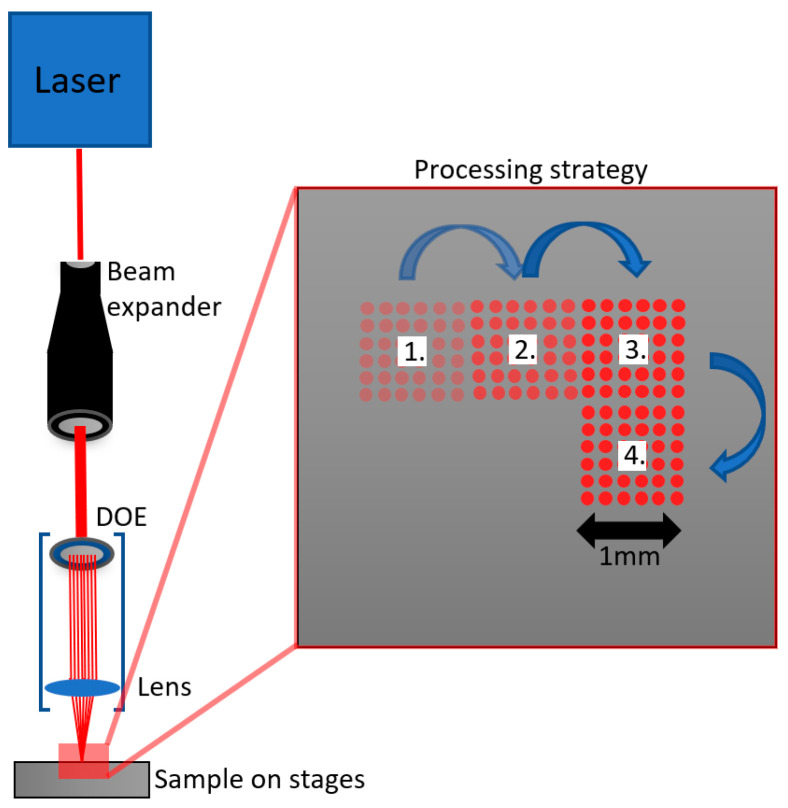
Simple schematics of optical and micromachining configuration together with the texturing approach. An advantageous square-shaped matrix of sub-beams allows the processing of larger areas without any overlap between matrixes.

AISI 316L stainless steel plates with a thickness of 5 mm were processed by a Ytterbium based diode-pumped solid-state laser system Perla (HiLASE, Dolní Břežany, Czech Republic) emitting 1.7 ps pulses with pulse energy up to 20 mJ at 1030 nm wavelength, the repetition rate of 1 kHz and M^2^ of 1.15. The incident beam was guided through the variable beam-expander into the beamsplitting and focusing optical system (Holo/Or Ltd., Ness Ziona, Israel) forming a square-shaped matrix of 201 × 201 sub-beams with a spot diameter of 4.9 µm in the image plane 40 mm lens. By using an additional axis system, it is possible to treat large samples by stitching the single pattern areas together. The simple schematics of optical configuration and texturing approach are depicted in Figure 1.

The fluence referred to in the experimental section was calculated as pulse energy of the whole matrix of sub-beams (measured at the sample surface) divided by the number of sub-beams and the area of spot diameter (π × 2.45^2^ µm^2^).

Immediately after laser texturing (in less than 15 min), samples were stored for 6 h in a vacuum chamber pumped down to 10^−4^ Pa by HiPace 80 turbopump with backing Duo 3 rotary vane pump (Pfeiffer Vacuum Technology AG, Aßlar, Germany) to promote favourable chemical changes and decrease the transition time to reach hydrophobic state [30]. After that, samples were stored in plastic box in atmospheric conditions.

The wettability was analysed for 24 h after the vacuum treatment by an optical contact angle measuring device OCA 15EC (Data Physics Instruments, Filderstadt, Germany). The static contact angle was analysed for a water droplet volume of 10 μL. The test results were acquired through an average of five measurements on different locations for every textured surface.

The surface morphology was investigated within a week after the laser processing by scanning electron microscope, FERA 3 (TESCAN, Brno, Czech Republic) at an electron energy of 5 kV and laser scanning confocal microscope, OLS5000 (Olympus, Tokyo, Japan).

### Beam Splitting with Diffractive Laser-Induced Texturing (DLITe) Elements

The operating principle of the DLITe patterning method used in this work was described by a previous article of the authors [31]. The DLITe splitter MS-835-J-Y-X (Holo/Or LTD, Ness Ziona, Israel) was designed to generate 201 × 201 diffractive orders with an order separation angle of 0.136 mrad. The splitter had a period of 7800 µm, and was designed to operate with a special custom-built wide-field focusing lens giving an order separation of 5 µm at EFL = 40 mm. Accordingly, beam size was set to be 12 mm, to achieve the desirable 0.65 ratio of period to beam size described in reference [31]. Under these operating conditions, the diffraction-limited spot size of the focuser was calculated to be 4.9 µm, resulting in dense patterning of the entire field covered by the splitter. This field was designed to be 1 mm × 1 mm square area, to facilitate effective stitching, and due to field limitations of the focus optic. Due to the high NA of the focuser (0.148), the Rayleigh length of the focused sub-beams was only 16 µm, resulting in strong sensitivity to defocus and tilts as in any high NA system. However, the perioding diffractive splitter itself has very little or no sensitivity to tolerances such as centring, input angle or input size, as it is a periodic phase element. Pattern uniformity of the 40,401 orders was simulated as <2.5% peak-to-valley contrast. However, due to the close packing of orders, mutual interference effects rule the uniformity, resulting in typical values of <15% peak to valley contrast, regardless of manufacturing tolerances. Such values were simulated for perfectly round beams with M2 = 1, real single-mode laser beams often have some ellipticity, resulting in typical values of <25% peak to valley contrast between the orders. 

## 3. Results and Discussion

**Figure 2:**
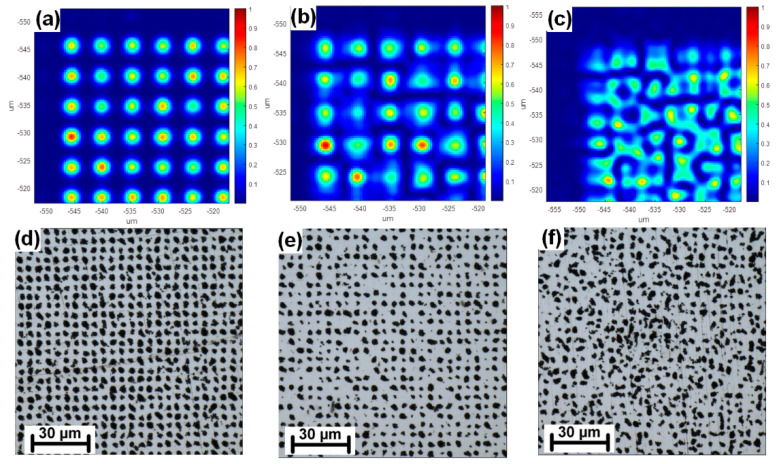
Example of defocused multi-beam pattern. (**a**) Simulation of optimal pattern shape; (**b**) Simulation of defocused pattern for 10 µm; (**c**) Simulation of defocused pattern for 20 µm; (**d**) Optimal pattern shape on a sample; (**e**) Defocused pattern for 10 µm on a sample surface; (**f**) Defocused pattern for 20 µm on a sample surface.

The combination of advanced beamsplitting element and tightly focused sub-beams requires precise alignment of all components with respect to the laser beam. Especially crucial is the distance between the splitting and focusing system and the sample surface (z-distance). A slight change in a z-distance for 10 µm already results in significant changes in pattern homogeneity. Figure 2 depicts the calculated change in a pattern homogeneity from the ideal shape (Figure 2a) to the defocused pattern for 10 µm (Figure 2b) and 20 µm (Figure 2c) and compares the calculation with experiment (Figure 2d–f).

**Figure 3:**
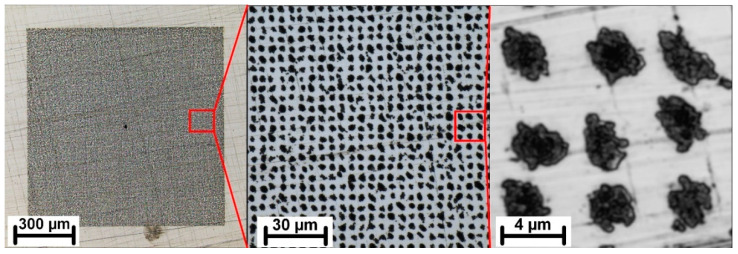
Detail of the best-quality pattern achieved by scanning the z-distance with 1 µm produced by 10,000 pulses with a peak fluence of 0.22 J/cm^2^.

Figure 3 shows a detail of the best-quality pattern achieved by scanning the z-distance with a step of 1 µm. The pattern still shows some inhomogeneities which might be improved in the future by applying a precise automated focusing system with sub-micron resolution.

**Figure 4:**
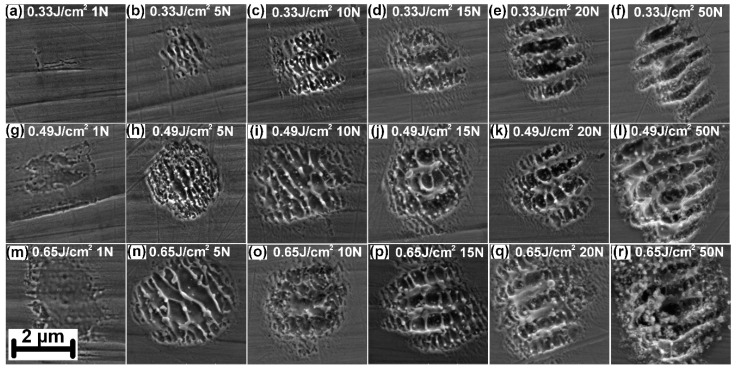
(**a**–**r**) Evolution of nanostructures formed inside each microcrater in a dependency on applied fluence and number of pulses (N).

In the following step, the formation of nanostructures was investigated with respect to a different number of pulses (N) in a range of 5–50 and pulse energies of 1 mJ, 1.5 mJ and 2 mJ, which corresponds to peak fluences of 0.33 J/cm^2^, 0.49 J/cm^2^ and 0.65 J/cm^2^ in each spot of the matrix. These experiments revealed a formation of periodic nanostructures formed inside each individual spot, as depicted in Figure 4.

As can be observed in Figure 4, different kinds of nanostructures can be produced within individual microholes. A significant change in morphology is observed especially with the growing number of pulses. For a single pulse and fluence up to 0.49 J/cm^2^, the surface starts to be modified and slightly disrupted increasing the initial roughness (Figure 4a,g). In the case of a higher fluence of 0.65 J/cm^2^, the surface appears to be smoothed by rapid melting and solidification of the thin surface layer (Figure 4m). With the increased number of pulses to 5, small periodic nanograting can be observed inside each spot (Figure 4b,h,n). The higher is the fluence, the greater is the periodicity of this nanograting and the more melt can be observed inside each spot. The nanograting periodicity was measured as 220 ± 15 nm for 0.33 J/cm^2^, 302 ± 21 nm for 0.49 J/cm^2^ and 470 ± 40 nm for 0.65 J/cm^2^. By increasing the number of pulses to 10, this periodicity increased to 229 ± 31 nm for 0.33 J/cm^2^ and 381 ± 48 nm for 0.49 J/cm^2^. In the case of the highest fluence of 0.49 J/cm^2^, the small nanograting is already disrupted (Figure 4o). Except for the nanograting, a formation of ripples with the periodicity of 720 ± 24 nm and perpendicular orientation to the laser polarization starts to be observed (Figure 4c,i,o). The reason for this behaviour might be found in heat accumulation during consecutive laser pulses, when the transient temperature of the material may reach the melting point [32,33]. In addition, Marangoni shear-generated convection could lead to hydrothermal waves and eventually to ripple formation [34]. As the number of pulses increase, ripples start to dominate and from 50 pulses there is no nanograting observed (Figure 4f,l,r). High fluence and a high number of pulses also results in a lot of accumulated heat and melting around the main periodic structures (Figure 4l,r). In the case of 0.65 J/cm^2^ and 50 pulses, the beginning of microdrilling process can be observed (Figure 4r).

**Figure 5:**
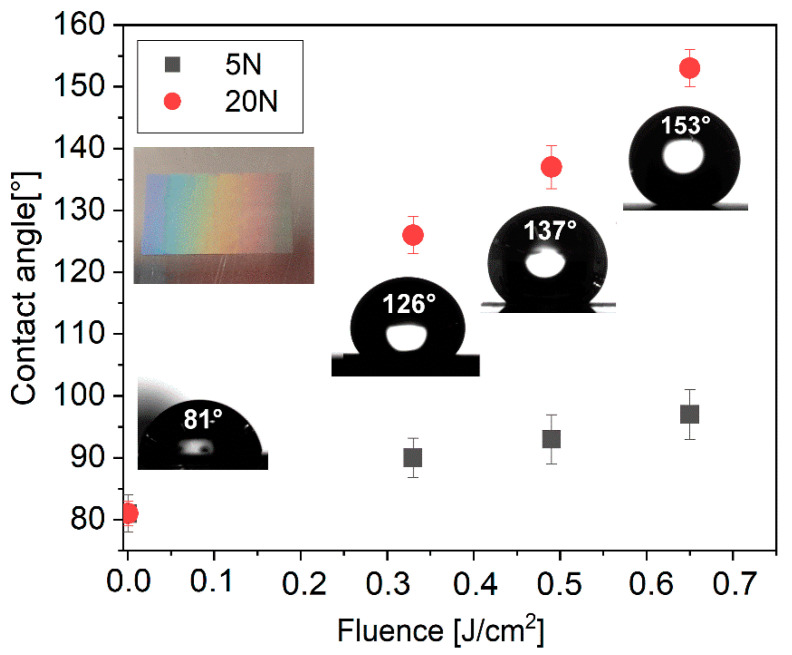
Contact angle evolution for samples stored in vacuum conditions for 6 h with insets of droplets on surface treated by 20 pulses and inset of the treated surface by 20 pulses demonstrating structural color due to diffraction on nanostructures. Fluence 0 J/cm^2^ depicts plane untreated surface stored in vacuum.

For the wettability analysis selected structures were produced on the area of 6 mm × 6 mm including structures depicted in Figure 4b,e,h,k,n,q which were produced by 5 and 20 pulses with a peak fluence of 0.33 J/cm^2^, 0.49 J/cm^2^ and 0.65 J/cm^2^. Immediately after laser treatment, samples show hydrophilic properties due to the formation of high-temperature metal oxides which have a high affinity towards the water molecules leading to hydrophilic behaviour [30]. Ageing in ambient conditions for several days is conventionally used to transfer samples from hydrophilic to hydrophobic state [35]. To speed up the transition samples were stored in a low-pressure environment containing hydrocarbons due to an oil-rotary vacuum pump [36]. As explained in our previous work [37], the dominant presence of non-polar hydrocarbons together with the absence of water molecules and convenient surface geometry is responsible for the superhydrophobic behaviour of the laser-treated sample stored in a low-pressure environment. As shown in Figure 5, samples stored for 6 h in vacuum conditions exhibit hydrophobic to superhydrophobic behaviour.

As can be observed in Figure 5, contact angle evolution is dependent on applied fluence and the number of pulses. Generally, higher fluence results in a larger spot, and thus, a higher percentage of the surface is textured with a fixed spot separation distance of 5 µm, which results in a higher contact angle. The type of structure also plays a significant role. Only a slight increase in contact angle from 81 ± 2° to 97 ± 4° is observed for shallow nanogratings fabricated by five consecutive laser pulses. On these surfaces, the droplets are pinned down with roll-off angles always above 20°. On the other hand, ripple structures exhibited a contact angle of 153 ± 3° with a roll-off angle of 7 ± 2.5° which can be considered as superhydrophobic surface.

The reason for different wettability of these two types of surfaces may be related to the depth of the surface features [30]. In the case of nanogratings, the depth is always below 80 nm which might have a minimal effect on a heavy 10 µL water droplet that may penetrate these shallow surface features. In contrast, ripple structures exhibit depth between 230–280 nm. Moreover, a higher number of pulses required for the ripple formation also results in a microcrater ablation with a diameter close to 4.9 µm and depth of ~1 µm for the superhydrophobic structure in Figure 4q. Hence the highest contact angle is reached for the deepest hierarchical structures composed of micro and nanoscale structures, which are often an optimal solution for a stable Cassie–Baxter state [9].

In addition to wettability, structural colour was observed on the processed sample surface as can be observed in the inset in Figure 5.

The advantageous square-shaped patterned area allows stitching each pattern with 0% overlap and thus significantly improve the throughput compared to the single beam approach. Within 5 ms for 5 consecutive laser pulses (repetition rate of 1 kHz), the simultaneous fabrication with 40,401 sub-beams allows production of 8,080,200 microcraters containing periodic nanograting (Figure 4h) per one second. In addition, this throughput may be easily up-scaled by increasing the repetition rate. Thus, in the future, this technique may demonstrate a great potential for rapid large-scale nano and micropatterning for the production of functional surfaces in industrial applications.

## 4. Conclusions

Fabrication of functional hydrophobic to superhydrophobic nanostructured surface has been demonstrated on stainless steel. Two different types of nanostructures have been produced with respect to periodicity and input beam polarization. For a low number of pulses around 5, nanogratings have been fabricated with periodicity below half of the input wavelength and with parallel orientation to the beam polarization. In addition, the periodicity of nanograting can be tailored by fluence. For a number of pulses above 10, ripples can be observed with a periodicity close to laser wavelength and perpendicular orientation to the beam polarization. The wettability of the surface can be tailored by following the structure geometry. Ripple structures demonstrated significantly better water repellence compared to shallow nanograting, reaching a contact angle up to 153 ± 3°. The combination of a high-energy pulsed picosecond laser system and the beam-splitting and focusing element allows simultaneous production of 40,401 nanostructured spots with a diameter below 5 µm ordered in a square-shaped matrix covering an area of 1 mm × 1 mm. By moving the stage, a processing rate of more than 8 million microcraters containing periodic nanograting per second can be achieved, significantly improving the throughput compared to the single beam approach and showing great potential for rapid large-scale surface functionalization.

## Data Availability

The data is available on reasonable request from the corresponding author.
